# Influence of blood thiosulfate produced by postmortem changes for the diagnosis of hydrogen sulfide poisoning in forensic autopsy

**DOI:** 10.2478/abm-2024-0035

**Published:** 2024-12-16

**Authors:** Masaaki Suzuka, Shigeki Jin, Akiko Takeuchi, Manabu Murakami, Keiko Takahashi, Kotaro Matoba

**Affiliations:** Department of Radiology, Graduate School of Dental Medicine, Hokkaido University, Hokkaido 060-8586, Japan; Department of Forensic Medicine, Graduate School of Medicine, Hokkaido University, Hokkaido 060-8638, Japan; Center for Cause of Death Investigation, Graduate School of Medicine, Hokkaido University, Hokkaido 060-8638, Japan; Center for Medical Education and International Relations, Faculty of Medicine, Hokkaido University, Hokkaido 060-8638, Japan

**Keywords:** hydrogen sulfide poisoning, liquid chromatography–tandem mass spectrometry, postmortem change, postmortem decomposition, thiosulfate

## Abstract

**Background:**

Thiosulfate concentration in blood is an important indicator for the diagnosis of hydrogen sulfide poisoning. It may also be detected at high levels in postmortem decomposition cases.

**Objectives:**

To determine the effect of postmortem decomposition on blood thiosulfate concentration and define precautions for diagnosing hydrogen sulfide poisoning based on thiosulfate concentration.

**Methods:**

A total of 57 cadavers (37 males and 20 females) of non-hydrogen sulfide poisoning-related deaths that underwent forensic autopsy in our department between 2016 and 2019 were classified into the non-decomposed (19 cases), partially decomposed (19 cases), and severely decomposed (19 cases) groups based on forensic findings. Blood samples collected from each case were analyzed for thiosulfate concentration using liquid chromatography–tandem mass spectrometry.

**Results:**

The mean concentration of thiosulfate detected in the blood was 70.9 (10.5–266.6) μmol/L in the severely decomposed group, 16.3 (0.1–52.7) μmol/L in the partially decomposed group, and 1.1 (0.1–3.6) μmol/L in the non-decomposed group. There was a statistically significant difference between each of the 3 groups (*P* < 0.01).

**Conclusions:**

Previous studies have reported a blood thiosulfate concentration of >14 μmol/L in hydrogen sulfide poisoning cases and <4 μmol/L in normal cases. Thus, thiosulfate concentration is believed to have a significant impact on the diagnosis of hydrogen sulfide poisoning. This study revealed that postmortem decomposition produced thiosulfate in the blood, and the concentration of thiosulfate was often as high as that observed in cases of hydrogen sulfide poisoning-related death. In addition to cases of advanced decomposition, an increase in thiosulfate concentration was also observed in cases of partial decomposition. Therefore, when measuring thiosulfate concentration as an indicator of hydrogen sulfide poisoning, it is necessary to carefully consider the influence of decomposition.

Thiosulfate, a metabolite of hydrogen sulfide, was first used in the 1980s as an indicator of hydrogen sulfide poisoning [1,2,3,4,5,6,7]. In the field of forensic science, gas chromatography–mass spectrometry (GC–MS) has long been used to measure thiosulfate [1]. However, its use for the measurement of large numbers of samples is restricted due to the influence of derivatization and other processes. We have recently developed a simple and sensitive method to measure thiosulfate using liquid chromatography–tandem mass spectrometry (LC–MS) [8,9,10].

Previous studies have reported that the concentration of hydrogen sulfide in blood and organs increases due to the effects of decomposition, regardless of the absence of hydrogen sulfide exposure [11,12,13]. By contrast, thiosulfate has been recognized as a stable indicator [1], and its concentration was >14 μmol/L in hydrogen sulfide poisoning deaths and <4 μmol/L in normal cases ]^1^, ^6^, ^7^, ^9^, ^14^,^15^,^16^,^17^,^18^]. However, two cases have been reported where high concentrations of thiosulfate were detected in the blood and organs of severely decomposed bodies, with causes of death other than hydrogen sulfide poisoning. Hence, it is necessary to consider the effects of decomposition when using thiosulfate to diagnose hydrogen sulfide poisoning [19].

Previous studies have not examined the extent to which decomposition generates thiosulfate. Therefore, it is necessary to measure the blood thiosulfate concentrations in decomposed cases, which are not associated with hydrogen sulfide poisoning, and to compare them with the blood thiosulfate concentrations in non-decomposed cases. In this study, we measured the blood thiosulfate concentrations of severely decomposed, partially decomposed, and non-decomposed forensic autopsy cases—whose cause of death was not hydrogen sulfide poisoning—using LC–MS method, which is highly sensitive and suitable for analyzing a large number of samples. The effect of postmortem decomposition on the blood thiosulfate concentration and precautions for diagnosing hydrogen sulfide poisoning based on thiosulfate concentration were examined.

## Methods

The data analyses were performed within the framework of routine medicolegal casework following the autopsy (2009) and ethical (2003) guidelines of the Japanese Society of Legal Medicine and approved by the ethics committee of Hokkaido University (No. 16-015). The need for informed consent was waived by our institutional ethics committee.

### Forensic samples

A total of 57 cadavers (37 males and 20 females; age: 2–92 [mean = 56.6] years) with non-hydrogen sulfide poisoning-related deaths (natural death, asphyxiation, carbon monoxide poisoning, drowning, freezing, and trauma deaths) that underwent judicial autopsy in our department between 2017 and 2019 were classified into non-decomposed (19 cases), partially decomposed (19 cases), and severely decomposed (19 cases) groups based on forensic findings; blood collected from the hearts were used as samples. The severely decomposed group included cases wherein putrefactive discoloration, putrefactive gas, and putrefactive marbling were observed throughout the body, while the partially decomposed group included cases wherein putrefactive discoloration, putrefactive gas, and putrefactive marbling were observed only in certain parts of the body, such as the abdomen. The non-decomposed group included cases wherein no decomposition phenomena were observed at all.

### Reagents

The following reagents were purchased: methanol, for high-performance liquid chromatography (Wako Pure Chemical Industries, Ltd., Osaka, Japan); formic acid, for LC–MS (Wako Pure Chemical Industries, Ltd.); 0.01 mol/L sodium thiosulfate solution (Wako Pure Chemical Industries, Ltd.); phenyl 4-hydroxybenzoate (Tokyo Chemical Industry Co., Ltd., Tokyo, Japan); pentafluorobenzyl bromide (Tokyo Chemical Industry Co., Ltd.); sodium chloride (special grade; Fujifilm Wako Pure Chemical Industries, Ltd.); and L-ascorbic acid (Tokyo Chemical Industry Co., Ltd.). Ultrapure water was prepared using the PURELAB flex (ELGA LabWater, Buckinghamshire, UK).

### LC–MS analysis

Thiosulfate concentrations were measured via LC–MS using a pentafluorobenzyl derivatization of thiosulfate. Details of sample preparation using an ultrafiltration membrane and the measurement method were described in a previous study [10]. The lower limit of detection (LLOD) and the lower limit of quantification (LLOQ) in the method were 0.05 μmol/L and 0.1 μmol/L, respectively.

### Statistical analysis

The Steel-Dwass test (*P* < 0.01) was performed to compare the measured values of the severely decomposed, partially decomposed, and non-decomposed groups. Additionally, a simple regression analysis was performed to determine whether there was a correlation between thiosulfate concentration and postmortem interval (PMI). Statistical analyses were performed using JMP (version 11.0.0; SAS Institute Inc., Cary, NC, USA).

## Results

The average PMI was 1.4 (0.2–3) days in the non-decomposed group, 3.7 (2–14) days in the partially decomposed group, and 41.6 (3–150) days in the severely decomposed group. The average blood thiosulfate concentration of the severely decomposed group was 70.9 (10.5–266.6) μmol/L, while that of the partially decomposed group was 16.3 (0.1–52.7) μmol/L, with many cases exceeding 14 μmol/L (**Tables 1 and 2**). The mean blood thiosulfate concentration was 1.1 (0.1–3.6) μmol/L in the non-decomposed group and <4 μmol/L for all cases in this group (**Tables 1 and 2**). There was a statistically significant difference between each of the three groups (*P* < 0.01).

**Table 1.** Baseline characteristics and thiosulfate concentration among the severely decomposed, partially decomposed, and non-composed groups

	**Severely decomposed group (n = 19)**	**Partially decomposed group (n = 19)**	**Non-decomposed group (n = 19)**
Males	12	14	11
Females	7	5	8
Age (years)	58.8 ± 20.0 [27–87]	58.0 ± 24.0 [19–86]	53.0 ± 25.0 [2–92]
PMI (days)	34.2 ± 41.6 [3–150]	6.1 ± 3.7 [2–14]	1.4 ± 0.7 [0.5–3]
TS conc. (μmol/L)	70.9 ± 84.6 [10.5–266.6]	16.3 ± 18.3 [0.1–52.7]	1.7 ± 1.1 [0.1–3.6]

PMI, postmortem interval; TS conc., thiosulfate concentration.

**Table 2.** The number of cases classified by thiosulfate concentration

	**Thiosulfate concentration range (μmol/L)**		
	**<4**	**4–14**	**>14**
Severely decomposed group	0	2	17
Partially decomposed group	7	4	8
Non-decomposed group	19	0	0

**Figure 1.**
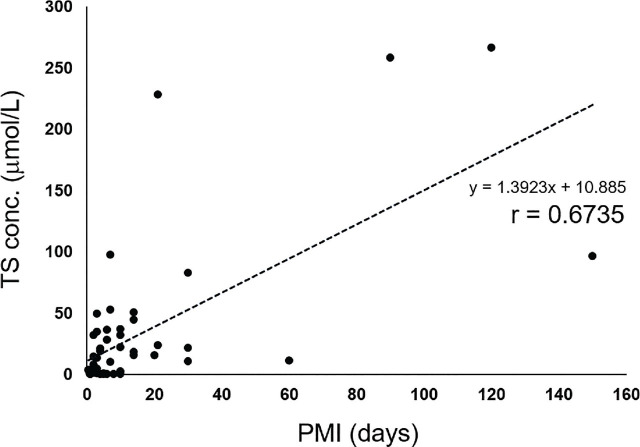
Correlation between PMI and TS conc. in all samples. PMI, postmortem interval; TS conc., thiosulfate concentration.

**Figure 2.**
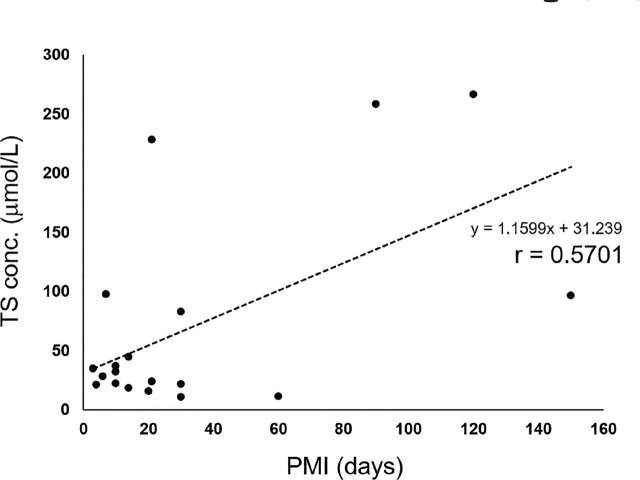
Correlation between PMI and TS conc. in the severely decomposed group. PMI, postmortem interval; TS conc., thiosulfate concentration.

**Figure 3.**
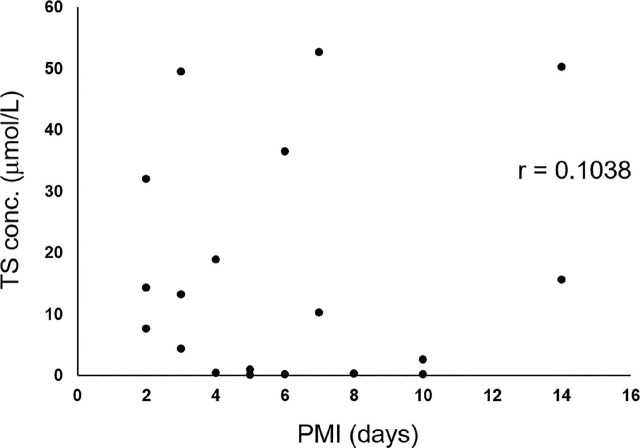
Correlation between PMI and TS conc. in the partially decomposed group. PMI, postmortem interval; TS conc., thiosulfate concentration.

To investigate whether any specific relationship exists between blood thiosulfate concentrations and PMI in all cases, an approximate equation (regression line)—with thiosulfate concentration on the vertical axis (*y*) and PMI on the horizontal axis (*x*)—was used as follows: *y* = 1.3923*x* + 10.885; the correlation coefficient was 0.6735, indicating a trend of increasing thiosulfate with increasing PMI (**Figure 1**). In the severely decomposed group, a regression line was obtained, with *y* = 1.1599*x* + 31.239; the correlation coefficient was 0.5701 (**Figure 2**). In the partially decomposed (**Figure 3**) and non-decomposed groups, a correlation between PMI and thiosulfate concentration was not observed.

## Discussion

The blood thiosulfate concentrations of healthy subjects have been reported to be extremely low. In a previous report by Kage et al. [1], the blood thiosulfate concentrations of 12 healthy subjects were below the detection limit (3 μmol/L) of the GC–MS method. Togawa et al. [7] reported an average of 0.61 (0.537–0.690) μmol/L thiosulfate in the serum of 5 healthy subjects, measured by high-performance liquid chromatography. Additionally, Kawanishi et al. [6] reported that the plasma thiosulfate concentrations of 5 healthy subjects were 2.41–3.04 (average: 2.68) μmol/L. These reports indicate that the blood thiosulfate concentration in healthy subjects was extremely low and did not exceed 4 μmol/L. The blood thiosulfate concentration of the non-decomposed group in this study was 0.1–3.6 (average: 1.1) μmol/L, and no cases had a concentration of >4 μmol/L. Therefore, cases with no evidence of decomposition would be expected to present low values, similar to those of healthy, living subjects. In the partially decomposed group, the values exceeded 4 μmol/L in 12/19 cases; in the severely decomposed group, all cases exceeded 4 μmol/L despite not being associated with hydrogen sulfide poisoning and therefore expected to present concentration of <4 μmol/L. From these results, it is clear that thiosulfate concentrations increase through postmortem decomposition, regardless of hydrogen sulfide poisoning. It is difficult to specify the timing of the postmortem increase in thiosulfate concentration; however, it is believed to rise when parts of the specimens decompose, with severely decomposed specimens undoubtedly generating thiosulfate postmortem.

There are many reports regarding thiosulfate levels in blood samples collected from cadavers with hydrogen sulfide poisoning. Maebashi et al. [14] reported thiosulfate concentrations ranging from 14 μmol/L to 648 μmol/L in the blood of 17 cadavers with hydrogen sulfide poisoning-related deaths. Kage et al. [15,16,17] reported 25–220 μmol/L thiosulfate concentrations in the blood of cadavers who died of hydrogen sulfide poisoning in factory effluent, underground wastewater tanks, and volcanic gases backflowing into a geothermal power plant. Moretti et al. [18] reported that the concentration of thiosulfate in the cardiac blood of four people who died of hydrogen sulfide poisoning in the same tank ranged from 18.4 μmol/L to 35.1 μmol/L. The results of these studies suggest that most cases of death from hydrogen sulfide poisoning involve blood thiosulfate concentrations of ≥14 μmol/L.

Miyazato et al. [19] measured thiosulfate concentrations in the organs and bodily fluids of four cadavers with hydrogen sulfide poisoning-related deaths, two decomposed cases with deaths from causes other than hydrogen sulfide poisoning, and two cases with no evidence of decomposition. In the two obviously decomposed cases, thiosulfate concentrations in the left and right heart blood were 87.7 μmol/L and 81.8 μmol/L at 7 days postmortem and 38.0 μmol/L and 29.0 μmol/L at 12 days postmortem, respectively. This study was the first to report that thiosulfate concentrations increased to the same level as observed in cases of hydrogen sulfide poisoning [19].

In this study, the blood thiosulfate concentration in the severely decomposed group (n = 19) without hydrogen sulfide poisoning ranged from 10.5 μmol/L to 266.6 μmol/L (**Table 1**), which was 3–66 times higher than the blood thiosulfate concentration in the non-decomposed group. In 17/19 cases in the severely decomposed group, the blood thiosulfate concentrations were similar to those observed for death due to hydrogen sulfide poisoning (>14 μmol/L; **Table 2**). Although the level of thiosulfate in blood is considered a useful diagnostic indicator of death by hydrogen sulfide poisoning, the significant elevation of thiosulfate due to severe decomposition in this study indicates that it is difficult to diagnose hydrogen sulfide poisoning based on blood thiosulfate concentration in cases with advanced stages of decomposition.

In 7/19 cases in the partially decomposed group had normal values, and 8 cases had values as high as those observed for hydrogen sulfide poisoning-related deaths; in particular, 2 cases exceeded the threshold (14 μmol/L) for death by poisoning within about 2 days after death. There are no clear objective findings to determine whether level of thiosulfate increases due to postmortem decomposition. Therefore, if a case exhibits even partially decomposed findings, it is necessary to consider the possibility that the blood thiosulfate level may have increased due to postmortem, and make an informed judgment.

Thiosulfate levels were <4 μmol/L in all the cases in the non-decomposed group (**Table 2**). However, Miyazato et al. [19] reported that among two non-decomposed controls (not hydrogen sulfide poisoning), thiosulfate concentrations in the left and right heart blood were 9.0 μmol/L and 11.0 μmol/L with a 24-h PMI and 1.6 μmol/L and 0.8 μmol/L with a 29-h PMI, respectively. Despite there being a control case in the non-decomposed group, one case exceeded the normal value of 4 μmol/L for healthy subjects. Although the thiosulfate concentration did not exceed 14 μmol/L—a criterion for the diagnosis of hydrogen sulfide poisoning—it should be considered that mild elevation of thiosulfate may be observed in some postmortem environments with short PMIs (24 h).

A positive correlation between PMI and thiosulfate concentration was observed on a plot of all samples (**Figure 1**). In the severely decomposed group, a correlation was observed (**Figure 2**); however, no correlation was observed in the partially decomposed group (**Figure 3**). In the severely decomposed group, the degree of decomposition increased due to PMI, and the thiosulfate concentration was also considered to have increased. In the partially decomposed group, the lack of correlation may be due to the length of the PMI being shorter than that of the severely decomposed group. In addition, the individual differences due to the cause of death and the environment of the cadaver place were large. Further study is needed to clarify this issue.

## Conclusion

Thiosulfate was found to be produced in the blood by postmortem decomposition. The elevation of blood thiosulfate concentration caused by postmortem decomposition is often as high as observed for death by hydrogen sulfide poisoning, which may have a significant impact on its diagnosis. Therefore, when measuring thiosulfate as an indicator of hydrogen sulfide poisoning, the effect of decomposition should be carefully considered.
